# Obesity, smoking, alcohol consumption and years lived with disability: a Sullivan life table approach

**DOI:** 10.1186/1471-2458-11-378

**Published:** 2011-05-24

**Authors:** Bart Klijs, Johan P Mackenbach, Anton E Kunst

**Affiliations:** 1 Department of Public Health, Erasmus MC, University Medical Centre, PO Box 2040, 3000 CA Rotterdam, The Netherlands; 2 Department of Public Health, Academic Medical Centre (AMC), University of Amsterdam, PO Box 22660, 1100 DD Amsterdam, The Netherlands

## Abstract

**Background:**

To avoid strong declines in the quality of life due to population ageing, and to ensure sustainability of the health care system, reductions in the burden of disability among elderly populations are urgently needed. Life style interventions may help to reduce the years lived with one or more disabilities, but it is not fully understood which life style factor has the largest potential for such reductions. Therefore, the primary aim of this paper is to compare the effect of BMI, smoking and alcohol consumption on life expectancy with disability, using the Sullivan life table method. A secondary aim is to assess potential improvement of the Sullivan method by using information on the association of disability with time to death.

**Methods:**

Data from the Dutch Permanent Survey of the Living Situation (POLS) 1997-1999 with mortality follow-up until 2006 (n = 6,446) were used. Using estimated relative mortality risks by risk factor exposure, separate life tables were constructed for groups defined in terms of BMI, smoking status and alcohol consumption. Logistic regression models were fitted to predict the prevalence of ADL and mobility disabilities in relationship to age and risk factor exposure. Using the Sullivan method, predicted age-specific prevalence rates were included in the life table to calculate years lived with disability at age 55. In further analysis we assessed whether adding information on time to death in both the regression models and the life table estimates would lead to substantive changes in the results.

**Results:**

Life expectancy at age 55 differed by 1.4 years among groups defined in terms of BMI, 4.0 years by smoking status, and 3.0 years by alcohol consumption. Years lived with disability differed by 2.8 years according to BMI, 0.2 years by smoking and 1.6 by alcohol consumption. Obese persons could expect to live more years with disability (5.9 years) than smokers (3.8 years) and drinkers (3.1 years). Employing information on time to death led to lower estimates of years lived with disability, and to smaller differences in these years according to BMI (2.1 years), alcohol (1.2 years), and smoking (0.1 years).

**Conclusions:**

Compared with smoking and drinking alcohol, obesity is most strongly associated with an increased risk of spending many years of life with disability. Although employing information on the relation of disability with time to death improves the precision of Sullivan life table estimates, the relative importance of risk factors remained unchanged.

## Background

Due to ageing of the populations, the burden of disability is likely to further increase in the next decades [1]. Persons with one or more disabilities often experience declines in the quality of life and have an increased need for health care services [2-5]. As elderly people often spend at least part of their life while having one or more disabilities, declines in the quality of life are commonly associated with old age, as well as substantial health care expenditures at the end of life [6-9]. To avoid strong declines in the quality of life and to ensure sustainability of the health care system, reductions in the burden of disability are urgently needed.

Such reductions may be achieved by interventions that help persons adopting more healthy lifestyles, as is suggested by studies showing associations between lifestyle factors and years lived with disability [10-16]. Although the results of these studies are promising, there are some caveats. That is, almost all of these studies analyzed one single risk factor in isolation and did not compare effects of risk factors [10,12,13,15,16]. Moreover, estimates of the effects of smoking were inconsistent and, to date, estimates for alcohol consumption are lacking [12,14]. Consequently, it is still not fully understood which factor has the largest potential for achieving reductions in years lived with disability. Therefore, the primary aim of the study was to compare the effect of BMI, smoking and alcohol consumption on the life expectancy with disability.

Two standard methods for calculating the life expectancy lived with disability are the Sullivan life table and the multistate life table. The latter method is considered as most appropriate for modelling risk factor and population health dynamics [17-20]. However, the multistate life table method requires data on disability incidence, which are often unavailable or too imprecise due to small numbers of cases [19]. For these situations, one has to recur to the Sullivan life table method. Commonly, the input to the Sullivan table consists of a series of age-specific disability prevalence rates, multiplied with a factor to quantify the effect of exposure to a risk factor. Recently, however, it was demonstrated that the prevalence of ADL disability is not simply a function of age (i.e. time since birth) but that it is even more strongly associated with approaching death (i.e. time to death) [21,22]. The occurrence of disability sharply increases in the about 10 last years of life, and especially in the 5 last years. Using information on disability occurrence in relationship to end of life could result in more realistic estimates of the occurrence of disability across the life course. Consequently, Sullivan life tables could gain in precision by employing this additional information [21,22]. A secondary aim of the study was to assess whether employing information on time to death in the Sullivan life table may lead to substantively different estimates of the relative importance of these risk factors.

## Methods

### Study population

The study population consisted of respondents to three successive years (1997-1999) of the POLS health interview survey, which was conducted by Statistics Netherlands. The survey was representative for the Dutch population excluding the institutionalized population. Information was collected through face to face interviews. From 1997-1999, 52 198 subjects were approached and the response was 58%. For our analyses we selected elderly subjects who were 55 years and older (n = 6,446) at the time of the survey. Mortality among these subjects was registered until 2006 through linkage with the Dutch causes of death registry. The mean BMI in the study population was 25.0 kg/m^2 ^and the mean number of alcohol consumptions per week was 6.0. Further characteristics are presented in table 1. The POLS surveys and mortality data are administered by Statistics Netherlands and Data Archiving and Networked Services (DANS; http://www.dans.knaw.nl/).

**Table 1:** Characteristics of the study population

	Number of respondents (%)	Mean age	Percentage males	Percentage married	Number disabled	Number of deaths
Normal weight	2814 (43.7)	67.4	46.9	69.9	293	762
Overweight	2699 (41.9)	66.7	53.5	72.8	297	673
Obese	704 (10.9)	66.2	37.2	66.5	147	179
Missing/other	229 (3.6)	72.3	23.3	48.5	68	113
						
Never smoker	2041 (31.7)	69.1	16.7	61.3	318	510
Former smoker	2686 (41.7)	67.0	64.4	77.1	289	714
Current smoker	1524 (23.6)	64.9	59.8	68.8	181	445
Missing	195 (3.0)	67.0	50.0	72.8	17	58
						
1-14 alc cons/wk	3937 (61.1)	66.5	52.2	74.2	330	917
>14 alc cons/wk	647 (10.0)	64.5	78.2	76.7	47	160
Non drinker	1859 (28.9)	69.5	27.6	58.7	428	650
Missing	3 (0.0)	66.0	33.3	100.0	0	0

### Disability

Respondents were asked if they were able to 'walk up and down the stairs', 'walk outside', 'enter/leave the house', 'sit down/get up from a chair', 'move around on the same floor', 'get in/out of bed', 'eat/drink', 'get dressed/undressed', 'wash face/hands' and 'wash completely'. For each item, respondents could answer with 'without difficulty', 'with minor difficulty', 'with major difficulty' and 'only with help'. We considered respondents disabled if they reported "with major difficulty" or "only with help" for at least one item.

### Risk factors

BMI was calculated as body weight/body length^2 ^and was classified as 'normal weight' 20-24.9 kg/m^2^; 'overweight' 25-29.9 kg/m^2^; and 'obesity' ≥30 kg/m^2^. On the basis of the questions 'do you smoke?' and 'did you smoke in the past?' subjects were classified as 'never smoker', 'former smoker' or 'current smoker'. A question 'do you ever drink alcohol?' and two questions asking for the number of alcoholic consumptions in the week and at weekends were used to construct the categories 'no drinker', '1-14 alcoholic consumptions per week' and 'more than 14 alcoholic consumptions per week'. Together with age and sex, we controlled for marital status ('married', 'divorced', 'widower' and 'never married') in all analyses. Further analyses revealed that control for educational level or household income level would not substantially change the results.

### Mortality analyses

Poisson regression methods were used to calculate the relative risk (RR) for mortality by BMI, smoking and alcohol. Univariate models were fitted that included dummy variables representing the different categories of BMI, smoking and alcohol, respectively. Normal weight, never smoker and 1-14 alcohol consumptions/week were chosen as reference categories. Missing values, and for BMI missing values and BMI<20 kg/m2, were treated as a separate group and were modelled in the regression models using dummy variables. Each model was adjusted for age (continuous), sex, and marital status. The RRs for mortality were used to calculate conversion factors that express the mortality level of exposed individuals in relationship to the average Dutch mortality levels. The conversion factors were applied to age-specific mortality rates for the Netherlands in the period 2000-2004 to construct separate life tables for each category of BMI, smoking and alcohol consumption.

### Disability analysis

Univariate logistic regression models were fitted in which disability (a dichotomous variable) was predicted as a function of risk factor exposure (dummies) and age (continuous). Sex and marital status were included to the models as control variables. Quadratic terms on age were significant and were therefore added to the prediction models. Interactions between age and risk factor exposure were not significant and were not included. The fitted models were used to predict the age specific prevalence of disability for each risk factor exposure category. In these predictions, the values of the control variables (sex and marital status) were set at the study population averages.

In further analysis, the age schedules of disability were not predicted on the basis of associations with age only, but also on the basis of associations with both age and time to death. For this analysis, regression models were fitted that were similar to the original models but also contained a variable measuring the time to death for each person that died during follow-up until 2006. Time to death was defined as the difference in time between the moment of the survey and the moment of death. Further details on this method, including ways to take into account survivors, are given elsewhere [21,22].

### Life table analysis

Sullivan life tables were constructed to calculate the years lived with disability (i.e. life expectancy with disability at age 55) for each risk factor exposure category [20]. For constructing these life tables, we utilized the estimated age schedules of mortality and disability, stratified by risk factor exposure category (see above).

In most analyses, the common version of the Sullivan life table method was applied. However, in the additional analyses aimed to take into account relationships between disability and time to death, a refined approach had to be taken. In this approach, annual age specific disability prevalences were estimated conditional on remaining years of life, and remaining years of life adjusted estimates of the age specific disability prevalence were used as input to the life table. As a first step, we stratified the life table population into subpopulations according to their age at death (or length of life). The number of people in subpopulation with length of life *x *was equal to the number who would die at age *x *according to the life table. Next, for each population with the same age at death, we estimated the age-specific schedule of disability, using the estimates of the logistic regression models described above. Finally, we estimated the age-specific prevalence rates of disability for the total life table population as the population-weighted sum of the age-specific prevalence rates in all subpopulations.

The total life expectancy and the years lived with disability were calculated for the aggregate life table population. The years lived without disability (i.e. disability free life expectancy) were calculated as the difference between the total life expectancy and the life expectancy with disability. Confidence intervals (CI) around the estimated life expectancy and years lived with and without disability were estimated using probabilistic sensitivity analyses [23-25]. That is, thousand times, regression coefficients were drawn randomly from each regression model, assuming multivariate normal distribution. For each draw, a life table was set up and total life expectancy and years lived with and without disability were calculated. The 25th and 975th of the ordered values indicated the boundaries of the CIs.

Regression analyses were performed using Stata 10.0 and life tables were constructed in Microsoft Excel 2002.

## Results

Of the three factors compared, smoking had the largest effect on mortality (table 2; RR current smoker: 1.62). The effects of alcohol consumption and BMI were substantially smaller (RR >14 cons/wk: 1.19; RR obese: 1.15, not significant).

**Table 2:** Relative risks for mortality and odds ratios for disability according to risk groups

	RR mortality with 95% CI	OR disability in models without time to death with 95% CI	OR disability in models with time to death with 95% CI
Normal weight	1.00	1.00	1.00
Overweight	0.97 (0.87-1.07)	1.24 (1.03-1.48)	1.26 (1.05-1.52)
Obese	1.15 (0.98-1.36)	2.73 (2.16-3.46)	2.76 (2.17-3.51)
			
Never smoker	1.00	1.00	1.00
Former smoker	1.18 (1.03-1.35)	1.25 (1.02-1.53)	1.19 (0.96-1.46)
Current smoker	1.62 (1.40-1.87)	1.58 (1.25-2.82)	1.36 (1.07-1.72)
			
1-14 alc cons/wk	1.00	1.00	1.00
>14 alc cons/wk	1.19 (1.00-1.41)	1.17 (0.84-1.62)	1.11 (0.80-1.56)
Non drinker	1.43 (1.29-1.59)	2.38 (2.01-2.82)	2.17 (1.83-2.58)

Separate models were fitted for BMI, smoking and alcohol consumption, but control for age, sex and marital status.

BMI had a substantial effect on the odds of disability (table 2; OR obese: 2.73). The effect of smoking was smaller but still significant (OR current smoker: 1.58), while the effect of drinking alcohol was not significant. In models that include time to death, the estimated odds ratios were slightly smaller for smoking and alcohol, but not for BMI.

**Figure 1:**
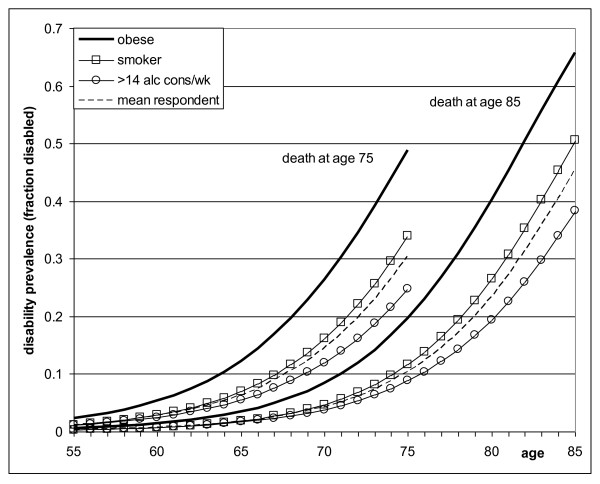
**Estimated prevalence of disability by age of death and according to risk factor**. Estimates were based on univariate models, controlled for sex and marital status.

Figure 1 estimates how the prevalence of disability increases during the last years of life. These estimates were derived from models that include both age and time to death. For persons who died at age 75, the prevalence of disability increased from below 0.05 to about 0.25 or 0.35 among drinkers and smokers, and to 0.50 among obese persons. Persons who died at an older age (i.e. age 85) had a different age pattern of disability. Their prevalence was about 60% lower at age 75, but towards the end of life, their chances of disability were substantially higher compared with younger decedents' chances at the end of life.

Table 3 shows the results of life table calculations. The difference in total life expectancy at age 55 was largest according to smoking (4.0 years between). The differences by alcohol consumption (3.0 years) and BMI (1.4 years) were smaller but were still substantial.

**Table 3:** Total life expectancy at age 55 and years lived with and without disability.

		Calculations without time to death		Calculations with time to death	
	Total life expectancy	Years without disability	Years with disability	Years without disability	Years with disability
Normal weight	26.0 (25.6 - 26.5)	22.9 (22.4 - 23.3)	3.2 (2.8 - 3.5)	23.8 (23.3 - 24.3)	2.3 (2.0 - 2.6)
Overweight	26.3 (25.8 - 26.8)	22.5 (22.0 - 23.0)	3.8 (3.4 - 4.3)	23.6 (23.0 - 24.1)	2.7 (2.4 - 3.1)
Obese	24.9 (23.8 - 26.1)	18.9 (17.9 - 19.9)	5.9 (5.1 - 6.9)	20.5 (19.5 - 21.6)	4.3 (3.7 - 5.0)
Variation by BMI	1.4 (0.1 - 2.9)	4.0 (2.8 - 5.2)	2.8 (1.9 - 3.8)	3.3 (1.9 - 4.4)	2.1 (1.4 - 2.7)
					
Never smoker	27.6 (26.9 - 28.5)	23.8 (23.1 - 24.5)	3.8 (3.4 - 4.4)	24.9 (24.2 - 25.7)	2.7 (2.3 - 3.1)
Former smoker	26.2 (25.8 - 26.8)	22.2 (21.7 - 22.7)	4.0 (3.6 - 4.5)	23.4 (22.8 - 23.9)	2.8 (2.5 - 3.2)
Current smoker	23.6 (23.1 - 24.3)	19.8 (19.2 - 20.4)	3.8 (3.3 - 4.4)	20.9 (20.3 - 21.5)	2.7 (2.3 - 3.2)
Variation by smoking	4.0 (2.8 - 5.2)	3.9 (2.9 - 4.9)	0.2 (-0.5 - 0.8)	4.0 (2.9 - 5.1)	0.1 (-0.4 - 0.6)
					
1-14 alc cons/wk	27.0 (26.7 - 27.4)	23.9 (23.5 - 24.3)	3.1 (2.8 - 3.5)	24.8 (24.3 - 25.2)	2.2 (1.9 - 2.6)
>14 alc cons/wk	25.6 (24.3 - 26.9)	22.5 (21.2 - 23.6)	3.1 (2.4 - 4.2)	23.3 (22.1 - 24.5)	2.3 (1.7 - 2.9)
Non drinker	24.1 (23.5 - 24.6)	19.3 (18.7 - 19.9)	4.8 (4.3 - 5.2)	20.6 (20.0 - 21.2)	3.4 (3.1 - 3.8)
Variation by alcohol	3.0 (2.1 - 3.9)	4.6 (3.8 - 5.4)	1.6 (1.0 - 2.2)	4.2 (3.3 - 5.0)	1.2 (0.8 - 1.6)

The difference between groups in years lived with disability was largest according to BMI (2.8 years between). The difference according to alcohol consumption (1.6 years) was smaller but still substantial and the difference according to smoking was only small (0.2 years). Obese persons spent a much longer period in disability (5.9 years), as compared to smokers and drinkers (3.8 and 3.1 years).

Estimates of the years lived with disability and differences according to risk factor were mostly smaller when calculated using regression models and life tables including time to death. According to this method the difference according to BMI was 2.1 years and according to smoking or alcohol consumption 0.1 and 1.2 years. Obese persons lived 4.3 years with disability, smokers 2.7 years and drinkers 2.3 years. The results regarding which factor was most important remained unchanged.

## Discussion

### Summary of results

The current paper is among the first to compare the effect of different life style factors on years lived with disability. Compared with smoking and drinking alcohol, obesity is more strongly associated with an increased risk of spending many years in disability during life. Using information on time to death in the Sullivan life table does not lead to substantively different estimates of the relative importance of the risk factors.

### Evaluation of data and methods

Among all subjects approached in the baseline survey, the non-response was 42%. We could not directly evaluate the effect of selective response on our estimates of years lived with disability, but the total mortality in our study sample (3.15% per year) was comparable to the total mortality in the Dutch population (1997-2006) aged 55 and older (3.21% per year) [26]. Although non-response is likely to lead to biased estimates of the prevalence of smoking and alcohol intake, it may not substantially affect associations between these risk factors and health outcomes [27]. None the less, we cannot exclude that selective non-response may have biased our estimates of effects of risk factors on years lived with disability.

The use of self reported measures of disability may have caused some reporting bias, particularly if risk factor exposure was related to reporting behaviour, independently from status of disability. However, in previous studies it was shown that self report of ADL disabilities correlates strongly with performance based measurement of disability [28], suggesting that it is unlikely that reporting bias could have substantially biased our results.

Self-reported BMI tends to be underreported, particularly by those who have a high BMI [29,30]. Therefore, the risk of disability among persons who had a high BMI may have been overestimated. However, the potential bias has been shown to be acceptable for correlation analyses like in our study [30]. Underreporting in self-reported alcohol consumption may affect prevalence rates, but does not appear to have substantially bias effect on the association between heavy drinking and harmful consequences [31].

The institutionalized population, an old population with a high disability prevalence, was not included in the baseline survey [32,33]. It cannot be excluded that this exclusion may have led to some bias in our estimates of risk factors in relation to years lived with disability. However, in the Netherlands, only a minor part of elderly people live in an institution, e.g. 90% of those aged 80-85 still live at home. Hence, the bias because of excluding the institutionalized population will probably be small [32,33].

Our choice of disability cut-off level was arbitrary. Using a more stringent cut-off level of having "with major difficulty" or "only with help" for at least two items resulted in (non-significantly) higher odds ratios for disability for BMI (OR obesity = 2.96) and alcohol consumption (OR >14 alc cons/wk = 1.52), but in a lower odds ratio for smoking (OR current smoker = 1.48). Years lived with disability differed by 2.0 years according to BMI, 0.2 years by smoking and 1.4 by alcohol consumption. Including time to death to the calculation yielded similar results. It can be concluded that the cut-off level to define disability has had no major influence on our substantive conclusions.

The association of BMI, smoking and alcohol with disability is likely to be mediated by the occurrence of specific diseases or other risk factors such as physical activity. If our aim had been to gain insight into causal chains that relate risk factor exposure with disability, it would have been useful to include more covariates. However, as our analysis had a descriptive purpose, and adjusting for co-morbidities or physical activities would take out part of the effect of lifestyle on disability, we used simple and transparent univariate regression models, adjusted for age, sex and marital status only, to estimate the years lived with and without disability.

### Comparison with previous studies

A few other studies compared lifestyle factors with respect to their effect on years lived with disability. Most studies used other outcome measures [14,34-36]. Comparable with our results, these studies found that, compared to heavy drinking or regular smoking, obesity had a much greater effect on the number of years that people could expect to live with long-standing illness, with reduced quality of life, or with less than good self-assessed health [34-36]. The only study that compared effects of different lifestyle factors on years lived with disability confirmed our key finding that obesity is more important than smoking [14].

### The obesity paradox

We found that smoking is associated with shorter life, whereas obesity is associated with spending more life years with disability. This difference is likely to be related to the fact that a high BMI is more strongly associated with non-lethal disabling diseases, such as osteoarthritis and chronic back pain, whereas smoking is more strongly associated with a series of fatal diseases with a relatively short period of disablement, such as lung cancer and other types of cancer [37-39].

Compared with other risk groups, obese persons on average spend a larger part of their last years of life with disability (Figure 1). This observation represents another side of the 'obesity paradox', which refers to the fact that increased BMI is an independent risk factor for heart failure, but that among patients with established heart failure, those who are overweight or obese are at decreased risk for death [40-44]. This suggests that obesity is a 'stretcher of disease and disability', which results in a high prevalence of disability prior to death among obese persons.

### Time to death in the Sullivan life table

The occurrence of ADL disability is not only associated with age (time since birth), but even more strongly with time to death [21,22]. A substantial part of disability occurs in relation with end-of-life processes [21,22]. As a result, a longer life is likely to be associated with a shift of the burden of disability towards older ages [21,22]. Conventional Sullivan life table methods do not account for possible shifts of disability towards older ages, but use one age schedule of disability, irrespective of the length of life (i.e. age of death) of individual people. Using an innovative approach, we accounted for the association of disability with length of life by defining schedules of disability not only as a function of age, but also as a function of age of death (Figure 1). The new estimates of the number of years lived with disability differed substantially from the original estimates. As expected, the expected years lived with disability were lower according to the new estimates. However, the relative importance of risk factors remained unchanged.

Therefore, this new methodology may be useful for obtaining more precise estimates of the occurrence of disability across the life cycle. It may be especially useful to assess the effect of increasing life expectancies on years with disability, which may have been overestimated in conventional methodologies. On the other hand, conventional methods appear to have yielded valid estimates of the relative importance of different risk factors.

## Conclusion

Of all risk factors, the variation in years lived with disability was largest for BMI. The largest reductions in the years that are spent with disability can in principle be achieved among obese people. Consequently, curtailing the obesity epidemic is urgently needed to prevent strong increases in the future burden of disability and to increase the prospects for healthy ageing for future generations of elderly.

## Competing interests

The authors declare that they have no competing interests.

## Authors' contributions

AEK and BK planned the study, developed and refined the methodological approach and discussed and, together with JPM, substantiated the interpretation of the results. BK performed all statistical analysis and wrote the paper. AEK helped revising the paper together with JPM. All authors read and approved the final manuscript.

## Pre-publication history

The pre-publication history for this paper can be accessed here:

http://www.biomedcentral.com/1471-2458/11/378/prepub

## References

[B1] Statistics Netherlands Statline databank\Bevolking\Prognose\Actuele prognose\Bevolkingsprognose 2008-2050 2010 Statistics Netherlands, the Hague/Heerlen

[B2] Fried TR Bradley EH Williams CS Tinetti ME Functional disability and health care expenditures for older persons Arch Intern Med 2001 161 21 2602 2607 10.1001/archinte.161.21.2602 11718592

[B3] Kovacs FMMDP Abraira VP Zamora JP Teresa Gil del Real MMPH Llobera JMDMPH Fernandez CMD the Kovacs-Atencion Primaria G Correlation Between Pain, Disability, and Quality of Life in Patients With Common Low Back Pain Spine 2004 29 2 206 210 10.1097/01.BRS.0000107235.47465.08 14722416

[B4] Verbrugge LM Patrick DL Seven chronic conditions: their impact on US adults' activity levels and use of medical services Am J Public Health 1995 85 2 173 182 10.2105/AJPH.85.2.173 7856776 PMC1615320

[B5] de Meijer CA Koopmanschap MA Koolman XH van Doorslaer EK The role of disability in explaining long-term care utilization Med Care 2009 47 11 1156 1163 10.1097/MLR.0b013e3181b69fa8 19786914

[B6] Zweifel P Felder S Meiers M Ageing of population and health care expenditure: a red herring? Health Econ 1999 8 6 485 496 10.1002/(SICI)1099-1050(199909)8:6<485::AID-HEC461>3.0.CO;2-4 10544314

[B7] Polder JJ Barendregt JJ van Oers H Health care costs in the last year of life--the Dutch experience Soc Sci Med 2006 63 7 1720 1731 10.1016/j.socscimed.2006.04.018 16781037

[B8] Seshamani M Gray AM A longitudinal study of the effects of age and time to death on hospital costs J Health Econ 2004 23 2 217 235 10.1016/j.jhealeco.2003.08.004 15019753

[B9] Werblow A Felder S Zweifel P Population ageing and health care expenditure: a school of 'red herrings'? Health Econ 2007 16 10 1109 1126 10.1002/hec.1213 17311357

[B10] Al Snih S Ottenbacher KJ Markides KS Kuo YF Eschbach K Goodwin JS The effect of obesity on disability vs mortality in older Americans Arch Intern Med 2007 167 8 774 780 10.1001/archinte.167.8.774 17452539

[B11] Ferrucci L Izmirlian G Leveille S Phillips CL Corti MC Brock DB Guralnik JM Smoking, physical activity, and active life expectancy Am J Epidemiol 1999 149 7 645 653 10192312 10.1093/oxfordjournals.aje.a009865

[B12] Nusselder WJ Looman CW Marang-van de Mheen PJ van de Mheen H Mackenbach JP Smoking and the compression of morbidity J Epidemiol Community Health 2000 54 8 566 574 10.1136/jech.54.8.566 10890867 PMC1731729

[B13] Peeters A Bonneux L Nusselder WJ De Laet C Barendregt JJ Adult obesity and the burden of disability throughout life Obes Res 2004 12 7 1145 1151 10.1038/oby.2004.143 15292479

[B14] Reuser M Bonneux LG Willekens FJ Smoking kills, obesity disables: a multistate approach of the US Health and Retirement Survey Obesity (Silver Spring) 2009 17 4 783 789 10.1038/oby.2008.640 19165165

[B15] Reynolds SL Saito Y Crimmins EM The impact of obesity on active life expectancy in older American men and women Gerontologist 2005 45 4 438 444 16051906 10.1093/geront/45.4.438

[B16] Walter S Kunst A Mackenbach J Hofman A Tiemeier H Mortality and disability: the effect of overweight and obesity Int J Obes (Lond) 2009 33 12 1410 1418 10.1038/ijo.2009.176 19786964

[B17] Mathers CD Robine JM How good is Sullivan's method for monitoring changes in population health expectancies? J Epidemiol Community Health 1997 51 1 80 86 10.1136/jech.51.1.80 9135793 PMC1060414

[B18] Rogers A Rogers RG Branch LG A multistate analysis of active life expectancy Public Health Rep 1989 104 3 222 226 2498971 PMC1579927

[B19] Robine J Jagger C Mathers C Crimmins E Suzman R Determining health expectancies 2003 West Sussex, UK: Wiley

[B20] Sullivan DF A single index of mortality and morbidity HSMHA health reports 1971 86 4 347 354 10.2307/4594169 5554262 PMC1937122

[B21] Klijs B Mackenbach JP Kunst AE Disability occurrence and proximity to death Disabil Rehabil 2010 32 21 1733 1741 10.3109/09638281003746049 20373858

[B22] Klijs B Mackenbach JP Kunst AE Future disability projections could be improved by connecting to the theory of a dynamic equilibrium Journal of Clinical Epidemiology 2010 in press 10.1016/j.jclinepi.2010.04.018 20800441

[B23] Boshuizen HC van Baal PH Probabilistic Sensitivity Analysis: Be a Bayesian Value Health 2009 10.1111/j.1524-4733.2009.00590.x 19695002

[B24] Briggs A Schulpter M Claxton K Decision Modelling for Health Economic Evaluation 2006 Oxford: Oxford University Press

[B25] Drummond M O'Brian B Stoddart G Methods for the economic evaluation of health care programs 2003 Oxford: Oxford University Press

[B26] European health expectancy monitoring unit (EHEMU) EHEMU database & information system Paris 201021611967

[B27] Van Loon AJ Tijhuis M Picavet HS Surtees PG Ormel J Survey non-response in the Netherlands: effects on prevalence estimates and associations Ann Epidemiol 2003 13 2 105 110 10.1016/S1047-2797(02)00257-0 12559669

[B28] Young Y Boyd CM Guralnik JM Fried LP Does self-reported function correspond to objective measures of functional impairment? J Am Med Dir Assoc 2010 11 9 645 653 10.1016/j.jamda.2009.12.084 21029999 PMC2966843

[B29] McAdams MA Van Dam RM Hu FB Comparison of self-reported and measured BMI as correlates of disease markers in US adults Obesity (Silver Spring) 2007 15 1 188 196 10.1038/oby.2007.504 17228047

[B30] Visscher TL Viet AL Kroesbergen IH Seidell JC Underreporting of BMI in adults and its effect on obesity prevalence estimations in the period 1998 to 2001 Obesity (Silver Spring) 2006 14 11 2054 2063 10.1038/oby.2006.240 17135623

[B31] Davis CG Thake J Vilhena N Social desirability biases in self-reported alcohol consumption and harms Addict Behav 35 4 302 311 10.1016/j.addbeh.2009.11.001 19932936

[B32] Klerk M Landelijk overzicht van de leefsituatie van oudere tehuisbewoners 2005 the Hague: The Netherlands Institute for Social Research

[B33] The Netherlands Institute for Social Research Rapportage ouderen 2006. Veranderingen in de leefsituatie en levensloop 2006 The Hague: The Netherlands Institute for Social Research

[B34] Bronnum-Hansen H Juel K Davidsen M Sorensen J Impact of selected risk factors on expected lifetime without long-standing, limiting illness in Denmark Prev Med 2007 45 1 49 53 10.1016/j.ypmed.2007.03.010 17467783

[B35] Bronnum-Hansen H Juel K Davidsen M Sorensen J Impact of selected risk factors on quality-adjusted life expectancy in Denmark Scand J Public Health 2007 35 5 510 515 10.1080/14034940701271908 17852988

[B36] Ostbye T Taylor DH The effect of smoking on years of healthy life (YHL) lost among middle-aged and older Americans Health Serv Res 2004 39 3 531 552 10.1111/j.1475-6773.2004.00243.x 15149477 PMC1361023

[B37] Kress AM Hartzel MC Peterson MR Burden of disease associated with overweight and obesity among U.S. military retirees and their dependents, aged 38-64, 2003 Prev Med 2005 41 1 63 69 10.1016/j.ypmed.2004.10.012 15916994

[B38] Must A Spadano J Coakley EH Field AE Colditz G Dietz WH The disease burden associated with overweight and obesity Jama 1999 282 16 1523 1529 10.1001/jama.282.16.1523 10546691

[B39] U.S. Department of Health and Human Services The health consequences of smoking: A report of the Surgeon General Rockville 2004

[B40] Kenchaiah S Evans JC Levy D Wilson PW Benjamin EJ Larson MG Kannel WB Vasan RS Obesity and the risk of heart failure N Engl J Med 2002 347 5 305 313 10.1056/NEJMoa020245 12151467

[B41] Bozkurt B Deswal A Obesity as a prognostic factor in chronic symptomatic heart failure American heart journal 2005 150 6 1233 1239 10.1016/j.ahj.2005.02.004 16338264

[B42] Curtis JP Selter JG Wang Y Rathore SS Jovin IS Jadbabaie F Kosiborod M Portnay EL Sokol SI Bader F Krumholz HM The obesity paradox: body mass index and outcomes in patients with heart failure Arch Intern Med 2005 165 1 55 61 10.1001/archinte.165.1.55 15642875

[B43] Kalantar-Zadeh K Block G Horwich T Fonarow GC Reverse epidemiology of conventional cardiovascular risk factors in patients with chronic heart failure Journal of the American College of Cardiology 2004 43 8 1439 1444 10.1016/j.jacc.2003.11.039 15093881

[B44] Gure TR Kabeto MU Blaum CS Langa KM Degree of disability and patterns of caregiving among older Americans with congestive heart failure J Gen Intern Med 2008 23 1 70 76 10.1007/s11606-007-0456-1 18030537 PMC2173919

